# Determinants of healthcare providers’ confidence in their clinical skills to deliver quality obstetric and newborn care in Uganda and Zambia

**DOI:** 10.1186/s12913-020-05410-3

**Published:** 2020-06-15

**Authors:** Min Kyung Kim, Catherine Arsenault, Lynn M. Atuyambe, Mubiana Macwan’gi, Margaret E. Kruk

**Affiliations:** 1 Department of Global Health and Population, Harvard T.H. Chan School of Public Health, 90 Smith Street, Boston, MA 02120 USA; 2 Department of Community Health and Behavioral Sciences, Makerere University School of Public Health, Kampala, Uganda; 3 Institute of Economic and Social Research, University of Zambia, Lusaka, Zambia

**Keywords:** Confidence, Healthcare provider, Knowledge, Newborn care, Obstetric care, Quality of care, Self-efficacy, Uganda, Zambia

## Abstract

**Background:**

Poor quality obstetric and newborn care persists in sub-Saharan Africa and weak provider competence is an important contributor. To be competent, providers need to be both knowledgeable and confident in their ability to perform necessary clinical actions. Confidence or self-efficacy has not been extensively studied but may be related to individuals’ knowledge, ability to practice their skills, and other modifiable factors. In this study, we investigated how knowledge and scope of practice are associated with provider confidence in delivering obstetric and newborn health services in Uganda and Zambia.

**Methods:**

This study was a secondary analysis of data from an obstetric and newborn care program implementation evaluation. Provider knowledge, scope of practice (completion of a series of obstetric tasks in the past 3 months) and confidence in delivering obstetric and newborn care were measured post intervention in intervention and comparison districts in Uganda and Zambia. We used multiple linear regression models to investigate the extent to which exposure to a wider range of clinical tasks associated with confidence, adjusting for facility and provider characteristics.

**Results:**

Of the 574 providers included in the study, 69% were female, 24% were nurses, and 6% were doctors. The mean confidence score was 71%. Providers’ mean knowledge score was 56% and they reported performing 57% of basic obstetric tasks in the past 3 months. In the adjusted model, providers who completed more than 69% of the obstetric tasks reported a 13-percentage point (95% CI 0.08, 0.17) higher confidence than providers who performed less than 50% of the tasks. Female providers and nurses were considerably less confident than males and doctors. Provider knowledge was moderately associated with provider confidence.

**Conclusions:**

Our study showed that scope of practice (the range of clinical tasks routinely performed by providers) is an important determinant of confidence. Ensuring that providers are exposed to a variety of services is crucial to support improvement in provider confidence and competence. Policies to improve provider confidence and pre-service training should also address differences by gender and by cadres.

## Background

The Lancet Commission on High Quality Health Systems revealed that the quality of obstetric and newborn health services remains poor and variable across low- and middle-income countries (LMICs) [1–4]. Inadequate healthcare provider competence has been identified as a contributing factor to the widespread poor quality care found in many LMICs [5–8]. Increasing care competence is one of the key strategy to improve health outcomes [4]. According to the Commission, competent care includes systematic patient assessments, correct diagnoses and the provision of appropriate treatments or preventive interventions, and proper patient counseling [4]. Thus, competent care begins with competent providers.

To be competent, providers need to be both knowledgeable and confident in their ability to perform clinical tasks. Inadequate knowledge is a key contributor to poor quality care in sub-Saharan Africa and several approaches have been used to improve knowledge including on-site training, mentoring, and supervision [9, 10]. But unlike knowledge, confidence has received little attention.

Confidence, also known as self-efficacy, is defined as a person’s beliefs in his/her capability to succeed in a specific situation or task [11, 12]. Several studies have shown that confidence is linked to provider behavior [13–15]. In one study from Indonesia, midwives with higher self-efficacy were more likely to perform newborn resuscitation attempts [14]. In the United States, confident physicians were more likely to adequately prescribe lipid-lowering medication to treat high cholesterol [15]. In addition, provider confidence contributes to job satisfaction and provider motivation which in turn, may be linked to how long the healthcare provider will remain in their current position [16–20].

Only a few studies have examined the determinants of provider confidence in obstetric and newborn care in LMICs [21]. The factors that lead to more confident healthcare providers remain unclear [10, 22, 23]. In addition, to our knowledge, no one has examined the links between actual knowledge, scope of practice (the range of clinical tasks routinely performed by providers) and confidence.

The aim of this study was to investigate the factors associated with levels of confidence among doctors, nurses, and other healthcare providers who performed obstetric and newborn care in Uganda and Zambia. In particular, we investigated the links between provider knowledge, recent scope of practice, and self-confidence in providing obstetric and newborn care. Findings from this analysis can provide insight into potentially modifiable mechanisms to increase provider confidence.

## Methods

### Study setting and sample

This study uses data from the implementation evaluation of the Saving Mothers and Giving Life (SMGL) intervention [24]. SMGL has significantly improved healthcare providers’ knowledge, confidence and job satisfaction [25]. This intervention was implemented in Uganda and Zambia from January – June 2012. For each country, four districts with a high maternal mortality ratio, a low facility delivery rate, and a high healthcare provider shortage were selected for implementation (Kabarole, Kamwenge, Kibaale, and Kyenjojo in Uganda and Mansa, Lundazi, Nyimba, and Kalomo in Zambia). These districts were primarily rural, with a largely agricultural workforce. The purpose of SMGL was to increase demand for and access to facility delivery and to improve the quality of care by including activities at both the health system and community levels. Healthcare providers received training on emergency obstetric care and newborn resuscitation. The core inputs and activities of the SMGL have been previously published [26].

The evaluation methods has been described elsewhere but in brief, it used a quasi-random post-test-only comparison group design [25]. The data for the evaluation were collected from May 2013 to July 2013. The evaluation team selected four comparison districts that were similar to the intervention districts in terms of geography, health system infrastructure, health system utilization, morbidity, and mortality. The comparison districts were Masindi and Kiryandongo in Uganda and Kapiri Mposhi and Kabwe in Zambia. Health facilities with the highest delivery volumes were selected in each district. These facilities included both rural and urban and at least one referral hospital which provides comprehensive emergency obstetric and newborn care.

### Instruments

The maternal healthcare providers (i.e. those clinicians directly involved in the provision of obstetric and newborn care such as delivery, antenatal care, and postnatal care) were invited to complete three questionnaires – a clinical confidence and scope of practice questionnaire, a job satisfaction questionnaire and an obstetric knowledge test. Eligible healthcare providers included all clinicians (i.e., doctors, nurses, midwives, nurse assistants, and clinical officers) who worked in maternity wards or provided obstetric and newborn care. All questionnaires with the exception of the knowledge test were conducted face-to-face; the test was self-administered. The knowledge test was adapted from one previously developed and validated by Johns Hopkins Program for International Education in Gynecology and Obstetrics (JHPIEGO) [27] a maternal health program based at Johns Hopkins University. This test included 60 multiple-choice questions on general obstetric knowledge for the management of early pregnancy, labor and delivery, and postpartum care. The knowledge test was developped based on World Health Organizations’ international guideline *Managing Complications in Pregnancy and Childbirth: A Guide for Midwives and Doctors* [28]. This guideline has been used throughout Africa, Asia, and the Americas [25]. Prior to start of data collection, the three instruments were pilot tested in non-study districts in Uganda and Zambia, and revised accordingly [25]. Providers’ demographics and facility characteristics were also included in the job satisfaction questionnaire.

### Measures

#### Confidence

We created a confidence score based on providers’ own assessment of their ability to perform 27 obstetric and newborn care tasks including for example, administering oxytocin, repairing perineal tears, and performing Kangaroo care (See Additional file 1 - Appendix E). The primary study [25] used a confidence score that included 26 tasks. For the present study, we decided to add two others “Newborn Apgar assessment” and “Kangaroo care” since these tasks are important component of immediate newborn care. We also removed “Administering anesthesia for C-sections” due to having only one anesthetist in our study sample (*N* = 1). Providers rated their confidence in being able to perform each task as very confident, not very confident, I cannot perform this skill, and does not apply. Providers who rated themselves as ‘very confident’ in performing a task were given one point per task and zero for ‘not very confident’ and ‘I cannot perform this skill’. Provider confidence was only assessed for tasks that each cadre believed they were expected to perform. ‘Does not apply’ responses were therefore treated as missing. For example, only general doctors, specialists, or medical licentiates [29] are expected to perform C-sections*.* The total score was converted into an average out of 100.

### Covariates

Scope of practice was measured by asking providers whether they had performed these same 27 tasks in the past 3 months. The scope of practice score was based on 27 tasks for doctors, obstetrics/gynecology specialist, and medical licentiates and 26 tasks for other cadres (performing C-sections was excluded for the other cadres). The total score was converted into a percentage.

The average provider knowledge score was calculated out of 100. We categorized the score into tertiles, after observing non-linearity in bivariate association between knowledge and confidence. The resulting categorical variable included providers with low (28–50%), average (51–58%), and high (60–93%) knowledge scores.

As with knowledge score, we categorized this into tertiles after observing non-linearity in the association between scope of practice and confidence. The resulting categorical variable included providers with low (0–48%), average (50–65%), and high (69–100%) levels of clinical practice in the past 3 months.

Other variables of interest included age, days of training in the past year, gender, qualification, the facility type, public or private ownership, whether the provider was part of the intervention or control group, and country. Age was treated as a continuous variable. A quadratic term for age was also included to account for non-linearity. Amount of training received in the past year was measured as the total number of days during which providers reported receiving on-site trainings.

There were seven cadres of providers in the study districts with different levels of training: 1) nurse assistant, 2) enrolled nurse, 3) enrolled midwife, 4) registered nurse, 5) registered midwife, 6) clinical officer and 7) general doctor, doctor specialists, and the medical licentiate. Nurse assistants are trained for about 6 months and exist only in Uganda. Enrolled nurses and enrolled midwives are trained for 2–3 years. Enrolled nurses are similar to licensed practical nurses in the United States. Registered nurses and registered midwives receive medical training for 3 to 4.5 years. Clinical officers receive 3 years of training. Doctors are typically trained for 5 to 7 years. Medical licentiates are clinical officers who received additional training so that they can perform the tasks that a doctor would typically perform [29]. Given the similar years of training, medical licentiates were included in the group of doctors.

Facility type was based on the availability of services at the facility and categorized into two groups. The first one included health centers that provide basic emergency obstetric and neonatal care (BEmONC). Seven signal functions are provided in BEmONC facilities: 1) Administration of parenteral antibiotics, 2) Administration of uterotonic drugs for active management of the third stage of labor and prevention of postpartum hemorrhage, 3) Use of parenteral anticonvulsants for the management of preeclampsia/eclampsia, 4) Manual removal of placenta, 5) Removal of retained products, 6) Assistance of vaginal delivery, and 7) Basic neonatal resuscitation [30]. The second category included hospitals that provide comprehensive obstetric and neonatal care (CEmONC) which perform the 7 basic functions of BEmONC and two additional services: cesarean delivery and blood transfusion [30]. Private ownership included both for-profit and not-for-profit facilities.

### Statistical analysis

We used a multiple linear regression model to investigate the factors associated with confidence, adjusting for covariates at the facility and provider levels. The unit of analysis was the provider and the regression model adjusted standard errors for clustering at the facility level. Three sensitivity analyses were performed. First, we added an interaction term between knowledge and scope of practice to observe whether the association between knowledge and confidence differed across levels of practice. Second, we regressed confidence on continuous measure of knowledge and scope of practice rather than tertiles. Third, we repeated the analysis in the intervention and the control facilities separately. All analyses were conducted in September 2019 using Stata SE version 16.0. *P*-values lower than 0.05 were considered statistically significant.

### Ethics

Since this study was a secondary analysis of de-identified data, it was not deemed to be human subjects research (NHSR) under the Harvard T.H. Chan School of Public Health Institutional Review Board (IRB) policy [31]. Thus, NHSR is exempt to acquire a formal IRB determination. The original study was approved by IRBs at Columbia University in the United States, Makerere University and the National Council for Science Technology in Uganda, and Excellence in Research Ethics and Science Converge Research Ethics Committee and Ministry of Health in Zambia [25]. Consent was obtained from the health care provider interviewed and the job satisfaction and knowledge surveys were completed in private rooms to ensure privacy. Data used for the study were stored in a secured folder with limited access.

## Results

In total, 577 maternal healthcare providers were included and 575 (99%) had all the data relevant for this analysis. One anesthetist was dropped from the analysis since he was the only provider in that cadre, leading to an analytical sample of 574 maternal healthcare providers.

The majority of providers were female, worked in public health centers, and enrolled midwives composed the largest group followed by enrolled nurses (Table 1). We found that on average, providers were confident in performing 71% of the surveyed tasks ranging from 0 to 100% (SD 20%). One provider was not confident in being able to perform any of the 27 tasks while 32 providers were confident in performing all of them. The average confidence score was higher among Zambian providers (74%) than Ugandan providers (69%).

**Table 1:** Characteristics of maternal healthcare providers and facilities in Uganda and Zambia

	Total	Uganda	Zambia
	(N = 574) N (%)	(*N* = 309) N (%)	(*N* = 265) N (%)
Confidence score (mean ± SD)	0.71 (0.20)	0.69 (0.20)	0.74 (0.20)
Knowledge score (mean ± SD)	0.56 (0.12)	0.53 (0.10)	0.59 (0.13)
Scope of practice (mean ± SD)	0.57 (0.24)	0.54 (0.24)	0.61 (0.23)
**Provider characteristics**			
Age (mean ± SD)	35.5 (10.5)	33.2 (10.1)	38.3 (10.3)
Days of training in past year (mean ± SD)	5.2 (10.8)	5.5 (12.2)	4.9 (8.8)
Female	398 (69.3%)	217 (70.2%)	181 (68.3%)
Qualification			
Nurse assistants^a^	15 (2.6%)	15 (4.9%)	–
Enrolled nurse^b^	140 (24.4%)	64 (20.7%)	76 (28.7%)
Enrolled midwife^c^	195 (34.0%)	96 (31.1%)	99 (37.4%)
Registered nurse^d^	45 (7.8%)	17 (5.5%)	28 (10.6%)
Registered midwife^e^	90 (15.7%)	58 (18.8%)	32 (12.1%)
Clinical officer^f^	56 (9.8%)	36 (11.7%)	20 (7.5%)
Doctor^g^	33 (5.7%)	23 (7.4%)	10 (3.8%)
**Facility characteristics**			
Facility level			
Health center^h^	339 (59.1%)	165 (53.4%)	174 (65.7%)
Hospital^i^	235 (40.9%)	144 (46.6%)	91 (34.3%)
Facility ownership			
Private (for profit, not-for profit)	153 (26.7%)	102 (33.0%)	51 (19.2%)
Public	421 (73.3%)	207 (67.0%)	214 (80.8%)
Facility location			
Rural	267 (46.5%)	141 (45.6%)	126 (47.5%)
Urban	307 (53.5%)	168 (54.4%)	139 (52.5%)
**Intervention district**			
Control	204 (35.5%)	113 (36.6%)	91 (34.3%)
Intervention	370 (64.5%)	196 (63.4%)	174 (65.7%)
**Country**			
Uganda	309 (53.8%)	309 (100%)	0 (0%)
Zambia	265 (46.2%)	0 (0%)	265 (100%)

Maternal healthcare providers are those clinicians who were directly involved in the provision of obstetric and newborn care such as delivery, antenatal care, and postnatal care. Confidence questionnaire was based on self-reported performance of 27 common obstetric tasks using four-level Likert item. Knowledge test was based on 60 multiple-choice questions on general obstetric knowledge for management of early pregnancy, labor and delivery, and postpartum care. Scope of practice questionnaire asked providers whether they had performed twenty-seven common obstetric tasks (the same as the confidence questionnaire) in the past three months. ^a^Nurse assistants are trained for about 6 months and exist only in Uganda. ^b^Enrolled nurses and ^c^enrolled midwives are trained for 2–3 years. Enrolled nurses are similar to licensed practical nurses in the United States. ^d^Registered nurses and ^e^registered midwives receive medical training for 3 to 4.5 years. ^f^Clinical officers receive 3 years of training. ^g^Doctors are typically trained for 5 to 7 years. Provider qualification can vary by country. ^h^Health centers are equipped to provide basic emergency obstetric and neonatal care (BEmONC) which includes 7 signal functions: 1) Administration of parenteral antibiotics, 2) Administration of uterotonic drugs for active management of the third stage of labor and prevention of postpartum hemorrhage, 3) Use of parenteral anticonvulsants for the management of preeclampsia/eclampsia, 4) Manual removal of placenta, 5) Removal of retained products, 6) Assistance of vaginal delivery, and 7) Basic neonatal resuscitation. ^i^Hospitals are equipped to provide comprehensive obstetric and neonatal care (CEmONC) which perform the 7 basic functions of BEmONC and two additional services: cesarean delivery and blood transfusion

The average scope of practice was 57% indicating that providers had performed 57% of the 27 surveyed tasks in the past 3 months on average (SD 24%). The scope of practice also ranged from 0 to 100%. The knowledge test revealed scores ranging from 28 to 93%, with a mean of 56%. Zambian providers scored 6% higher on the knowledge test than Uganda providers. Overall, confidence tended to be higher than knowledge and scope of recent clinical practice. Table 2 shows the average confidence, knowledge and scope of practice among the different provider cadre. Nurse assistants had the lowest confidence and knowledge while doctors scored highest on all three scores.

**Table 2:** Average confidence, knowledge, and scope of practice scores across maternal healthcare provider cadres in Uganda and Zambia, *N* = 574

Qualification	Confidence	Knowledge	Scope of practice
Nurse assistant^a^ (mean ± SD)	0.46 (0.22)	0.39 (0.09)	0.48 (0.16)
Enrolled nurse^b^ (mean ± SD)	0.65 (0.20)	0.51 (0.10)	0.50 (0.21)
Enrolled midwife^c^ (mean ± SD)	0.76 (0.15)	0.57 (0.12)	0.64 (0.20)
Registered nurse^d^ (mean ± SD)	0.67 (0.21)	0.59 (0.14)	0.45 (0.27)
Registered midwife^e^ (mean ± SD)	0.74 (0.18)	0.59 (0.08)	0.63 (0.26)
Clinical officer^f^ (mean ± SD)	0.64 (0.25)	0.53 (0.12)	0.43 (0.25)
Doctor^g^ (mean ± SD)	0.89 (0.14)	0.69 (0.10)	0.74 (0.23)
Average (N = 574)	0.71 (0.20)	0.56 (0.12)	0.57 (0.24)

Maternal healthcare providers are those clinicians who were directly involved in the provision of obstetric and newborn care such as delivery, antenatal care, and postnatal care. Confidence was measured by giving one point that providers rated themselves as ‘very confident’ in performing the clinical tasks. Knowledge was measured by summing all the correct answers on the 60-questionnaire test. Scope of practice was measured by assigning one point per clinical tasks that was performed in the past 3 months. All three scores were converted into average out of 100. ^a^Nurse assistants are trained for about 6 months and exist only in Uganda. ^b^Enrolled nurses and ^c^enrolled midwives are trained for 2–3 years. Enrolled nurses are similar to licensed practical nurses in the United States. ^d^Registered nurses and ^e^registered midwives receive medical training for 3 to 4.5 years. ^f^Clinical officers receive 3 years of training. ^g^Doctors are typically trained for 5 to 7 years. Provider qualification can vary by country

**Fig. 1:**
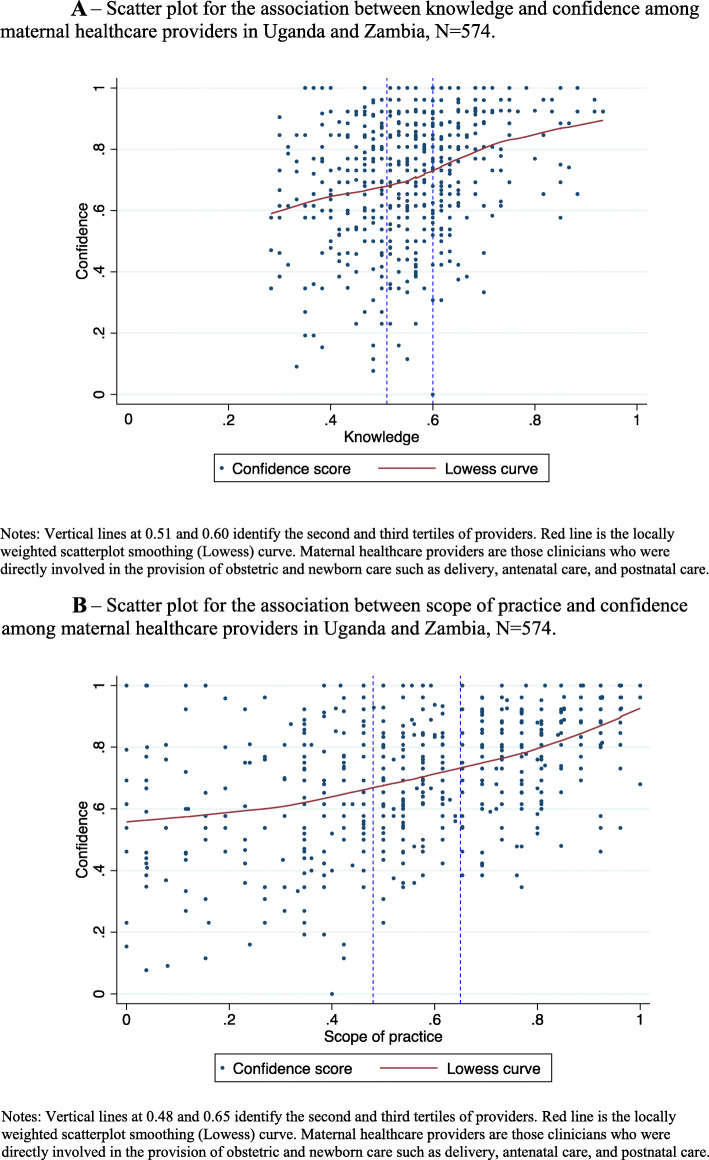
**a** Scatter plot for the association between knowledge and confidence among maternal healthcare providers in Uganda and Zambia, *N* = 574. **b** Scatter plot for the association between scope of practice and confidence among maternal healthcare providers in Uganda and Zambia, *N* = 574

Figure 1a shows continuous knowledge and confidence among the 574 providers, with two vertical lines identifying the second and third knowledge tertiles. Figure 1b plots continuous scope of practice and confidence among the 574 providers, with two vertical lines identifying the second and third tertiles of recent clinical practice. In both figures, the locally weighted scatterplot smoothing (lowess) curve shows a positive unadjusted association between knowledge and confidence and scope of practice and confidence, respectively.

Table 3 shows the results from the multiple linear regression. In the adjusted model, knowledge test scores were only moderately associated with confidence. Providers who scored between 60 and 93% on the test had a 4 percentage-point (95% CI 0.00, 0.08) higher confidence than those who scored less than 50%. We also found that being in second and third tertiles of scope of practice (i.e. performing more than 50% of expected clinical tasks in the past 3 months) was associated with a 5- and 13-percentage point increase in the confidence score compared to providers who practiced less than 50% of the tasks. Being a female was also associated with lower confidence compared to male counterparts after adjusting for cadre, age, and other covariates. Among cadres, enrolled midwives, registered midwives and doctors were associated with higher confidence scores compared to enrolled nurses (*p*-value < 0.05). In contrast, nurse assistants were associated with a 14-percentage point (95% CI -0.25, − 0.03) lower confidence compared to enrolled nurses. Enrolled nurses can be advanced to registered nurses by receiving an additional 1 to 1.5 years of training. Still in the adjusted model, being in public facilities and in the intervention districts also had small positive associations with confidence. Country was not associated with levels of provider confidence.

**Table 3:** Multiple linear regression for the association between provider and facility characteristics and confidence among maternal healthcare providers in Uganda and Zambia, *N* = 574

	Coefficient	95% CI	*P*-value
**Knowledge (ref. Knowledge score 28–50%)**			
51–58% Knowledge score	0.00	[−0.04, 0.04]	0.97
60–93% Knowledge score	0.04	[0.00, 0.08]	0.08
**Scope of practice (ref. Scope of practice 0–48%)**			
50–65% Scope of practice	0.05*	[0.01, 0.09]	0.01
69–100% Scope of practice	0.13**	[0.08, 0.17]	0.00
**Provider characteristics**			
Age	0.00	[−0.01, 0.01]	0.97
Age^2^	0.00	[0.00, 0.00]	0.77
Female	−0.05*	[− 0.09, 0.00]	0.03
Training received in days	0.00	[0.00, 0.00]	0.18
Qualification (ref. Enrolled nurse^a^)			
Nurse assistant^b^	−0.14*	[− 0.25, − 0.03]	0.01
Enrolled midwife^c^	0.08**	[0.05, 0.12]	0.00
Registered nurse^d^	0.03	[−0.03, 0.09]	0.35
Registered midwife^e^	0.06*	[0.01, 0.12]	0.02
Clinical officer^f^	−0.02	[−0.10, 0.05]	0.54
Doctor^g^	0.15**	[0.07, 0.24]	0.00
**Facility characteristics**			
Hospital^h^ (ref. Health center^i^)	−0.02	[−0.06, 0.01]	0.23
Public facility (ref. Private facility^j^)	0.03	[0.00, 0.07]	0.06
**Intervention district (ref. Control)**	0.03	[0.00, 0.07]	0.07
**Zambia (ref. Uganda)**	0.02	[−0.03, 0.06]	0.50

*N* = 574, R-squared = 28.20%. **p < 0.01, *p < 0.05. Outcome variable is a continuous variable for confidence levels. Standard errors are adjusted for clustering with facilities. Maternal healthcare providers are those clinicians who were directly involved in the provision of obstetric and newborn care such as delivery, antenatal care, and postnatal care. ^a^Enrolled nurses are trained for 2–3 years and are similar to licensed practical nurses in the United States ^b^Nurse assistants are trained for about 6 months and exist only in Uganda. ^c^Enrolled midwives are trained for 2–3 years. ^d^Registered nurses and ^e^registered midwives receive medical training for 3 to 4.5 years. ^f^Clinical officers receive 3 years of training. ^g^Doctors are typically trained for 5 to 7 years. Provider qualification can vary by country. ^h^Hospitals are equipped to provide comprehensive obstetric and neonatal care (CEmONC) which perform the 7 basic functions of BEmONC and two additional services: cesarean delivery and blood transfusion. ^i^Health centers are equipped to provide basic emergency obstetric and neonatal care (BEmONC) which includes 7 signal functions: 1) Administration of parenteral antibiotics, 2) Administration of uterotonic drugs for active management of the third stage of labor and prevention of postpartum hemorrhage, 3) Use of parenteral anticonvulsants for the management of preeclampsia/eclampsia, 4) Manual removal of placenta, 5) Removal of retained products, 6) Assistance of vaginal delivery, and 7) Basic neonatal resuscitation. ^j^Private facility includes both for-profit and not-for profit

The interaction term between knowledge and scope of practice was not significant (See Additional file 1 - Appendix A). Thus, the association between knowledge and confidence did not differ across levels of recent scope of practice. Similar results were found when we treated knowledge and confidence as continuous variables whereby the scope of practice was a stronger predictor of confidence than knowledge (See Additional file 1 - Appendix B). When we divided the samples into intervention and control groups, scope of practice remained a stronger predictor for the provider confidence than knowledge (See Additional file 1 - Appendix C and D).

## Discussion

Using data from Uganda and Zambia, we found a series of factors associated with healthcare provider confidence. Having recently performed a greater number of obstetric and newborn tasks was associated with greater provider confidence after adjusting for cadre, level of knowledge, training, age, and other factors. We also found that female health providers tended to be less confident than their male counterparts and that confidence levels varied greatly among different provider cadres with similar length of training. In contrast, knowledge scores were not strongly associated with confidence.

Overall, these findings have important implications for designing programs and policies to improve health providers confidence. More confident health providers tend to provide higher quality care and show greater satisfaction and retention [16–20]. To improve provider competency, previous studies have been largely focused on improving provider knowledge alone [32–34]. However, policymakers should also consider increasing the scope of practice to promote greater self-efficacy. These policies should also pay particular attention to differences in confidence by gender and cadre.

The positive association between scope of practice and confidence reveals that exposure to a variety of patients and tasks is an important contributor of provider confidence. This finding has implications for improving provider competence especially in rural clinics where patient volumes may be low. Although not directly linked to scope of practice, other studies have found a similar association between patient volumes and provider confidence [10, 22]. Rotating rural healthcare providers to higher volume facilities could be considered as a means to improve their confidence and competence. Furthermore, important variation in confidence across cadres might be a signal that lower-level providers are not getting enough exposure to common clinical tasks. Since the number of doctors in rural posts are often low, ensuring that lower-level providers are confident enough to perform these tasks is important to sustain the quality of care that mothers and newborns receive. In-service training could be another way to increase the confidence for these lower-level providers. In South Korea, nurses who treated a greater variety of patients also reported higher confidence in performing core clinical skills [35].

Gender was also significantly associated with provider self-efficacy in performing obstetric and newborn care, whereby female providers reported lower confidence compared to their male counterparts even after adjustment for cadre, age and other covariates. Perhaps this difference in confidence between genders could be partially driven by an unequal distribution of gender across cadres in our study. Almost all of the doctors (97%) in our sample were male while more than 90% of enrolled midwives were female. Nonetheless, studies of healthcare providers in Nigeria and medical students in America also found that females had much lower levels of confidence than males [36, 37]. Feminization of the healthcare workforce in LMICs is underway, especially in the field of gynecology [38, 39]. Policy makers need to pay attention to this shift in gender and take appropriate measures in medical training and in the workplace.

There was also substantial variation in confidence across different cadres. For example, despite receiving similar years of training, enrolled midwives reported significantly higher confidence than enrolled nurses even after adjusting for scope of practice. These differences may be explained by the type of pre-service training received by difference cadres. In Uganda, a review of curricula from 22 health professional training institutions showed that lectures and oral and written assignments were the predominant instruction methods [40]. One way to increase nurses confidence may be to provide competency-based pre-service medical education that includes clinical simulations and role play [41]. Midwives who generally receive competency-based education reported the same level of confidence in performing newborn resuscitation as doctors [41]. A systematic review by Bvumbwe et al. identified competency-based education as a key strategy to improve nursing education in Sub Saharan Africa [42].

Perhaps surprisingly, knowledge scores were not strongly associated with confidence. Scoring in the highest knowledge tertile (those who scored 60–93% on the test) was associated with only a 4% increase in confidence compared to scoring in the bottom tertile; and this association was not statistically significant (*p* = 0.08). It is possible that a certain threshold of knowledge score needs to be reached before it affects a provider’s belief in their ability to perform obstetric tasks. In contrast in a study in Nigeria, healthcare providers (doctors, nurses, and midwives) that scored higher on a knowledge assessment were significantly more likely to report higher confidence in performing emergency obstetrics care tasks compared to providers with lower scores [36]. Knowledge scores in our study were particularly low; only 4.4% of providers obtained a passing grade (> 80%) on the test.

This study had several limitations. First, our findings are limited to obstetric and newborn care which limits generalizability and may not apply to other clinical settings. Second, our sample only included a small number of doctors (*N* = 33, 5.9%) and nurse assistants (*N* = 15, 2.6%); therefore, the results for these cadres should be interpreted with caution. It is common to find a low number of doctors especially in LMICs and nurse assistants exist only in Uganda. Third, our data were collected at one time-point (i.e. cross-sectional) and thus our findings should not be interpreted as causal. Fourth, the findings are applicable only to the study countries. Lastly, our regression model only explained only 28% of the variation in providers’ confidence (adjusted R-squared = 28.2%). Other factors, including the quality of facility infrastructure, the availability of functional equipment and essential medicines and supplies, continuing education program, and adequate supervision systems could affect levels of providers’ confidence [13, 14].

Future research should explore factors associated with provider confidence in other clinical settings. Research on changes in provider confidence over time should also be conducted. Increasing provider confidence is important, but maintaining it should also be studied. Future studies should investigate associations between work environment and provider confidence to obtain a more comprehensive picture of factors that may improve provider confidence.

## Conclusions

Achieving the health-related sustainable development goals will require that health facilities in countries such as Zambia, Uganda, and other LMICs are staffed with adequate numbers of healthcare providers who are knowledgeable and confident in their ability to provide high-quality obstetric and newborn health services. Pre-service training as well as the clinical environment that provides adequate exposure to the full scope of care are critical ingredients in building a competent healthcare workforce.

## Supplementary information

**Additional file 1.**

## Data Availability

The datasets used and/or analyzed during the current study are available from the corresponding author on reasonable request.

## Abbreviations

LMICs: Low- and middle-income countries
SMGL: Saving Mothers and Giving Life
BEmONC: Basic emergency obstetric and neonatal care
CEmONC: Comprehensive obstetric and neonatal care
NHSR: Non-Human Subjects Research
IRB: Institutional Review Board
