# Supplementary material for: Determinants of healthcare providers’ confidence in their clinical skills to deliver quality obstetric and newborn care in Uganda and Zambia
Source: BMC Health Serv Res. 2020 Jun 15;20:539. doi: 10.1186/s12913-020-05410-3 (PMC7296707; doi:10.1186/s12913-020-05410-3)
Supplement: Supplementary file 1 — Additional file 1. [file 12913_2020_5410_MOESM1_ESM.docx]

## **Appendix A** – Linear regression for the association between provider and facility characteristics and confidence including an interaction term between knowledge and the scope of practice among maternal healthcare providers in Uganda and Zambia, N=574.

|  | Coefficient |  | 95% CI |  | P-value |
| --- | --- | --- | --- | --- | --- |
| **Knowledge (ref. Knowledge score 28% - 50%)** |  |  |  |  |  |
| 51% - 58% Knowledge score | -0.01 |  | [-0.08, 0.06] |  | 0.94 |
| 60% - 93% Knowledge score | 0.06 |  | [-0.03, 0.14] |  | 0.21 |
| **Scope of practice (ref. Scope of practice 0% - 48%)** |  |  |  |  |  |
| 50% - 65% Scope of practice | 0.06 |  | [-0.00, 0.13] |  | 0.06 |
| 69% - 100% Scope of practice | 0.13** |  | [0.06, 0.21] |  | 0.00 |
| **Knowledge and scope of practice interaction term** |  |  |  |  |  |
| 51%-58%#50%-65% | 0.01 |  | [-0.07, 0.10] |  | 0.76 |
| 51%-58%#69%-100% | -0.01 |  | [-0.10, 0.09] |  | 0.84 |
| 60%-93%#50%-65% | -0.04 |  | [-0.13, 0.06] |  | 0.46 |
| 60%-93%#69%-100% | -0.01 |  | [-0.13, 0.09] |  | 0.74 |
| **Provider characteristics** |  |  |  |  |  |
| Age | 0.00 |  | [-0.01, 0.01] |  | 0.96 |
| Age^2^ | 0.00 |  | [0.00, 0.00] |  | 0.76 |
| Female | -0.05* |  | [-0.10, -0.01] |  | 0.02 |
| Training received in days | 0.00 |  | [0.00, 0.00] |  | 0.20 |
| Qualification (ref. Enrolled nurse^a^) |  |  |  |  |  |
| Nurse assistant^b^ | -0.14* |  | [-0.25, -0.03] |  | 0.02 |
| Enrolled midwife^c^ | 0.08** |  | [0.04, 0.13] |  | 0.00 |
| Registered nurse^d^ | 0.03 |  | [-0.03, 0.10] |  | 0.36 |
| Registered midwife^e^ | 0.06* |  | [0.01, 0.12] |  | 0.02 |
| Clinical officer^f^ | -0.03 |  | [-0.10, 0.05] |  | 0.52 |
| Doctor^g^ | 0.15** |  | [0.07, 0.23] |  | 0.00 |
| **Facility characteristics** |  |  |  |  |  |
| Hospital^h^ (ref. Health center^i^) | -0.02 |  | [-0.06, 0.04] |  | 0.24 |
| Public facility (ref. Private facility^j^) | 0.03 |  | [0.00, 0.06] |  | 0.06 |
| **Intervention district (ref. Control)** | 0.03 |  | [-0.01, 0.07] |  | 0.08 |
| **Zambia (ref. Uganda)** | 0.02 |  | [-0.03, 0.06] |  | 0.49 |

Notes: N= 574. **p<0.01, *p<0.05. Outcome variable is a continuous variable for confidence levels. Standard errors are adjusted for clustering with facilities. Maternal healthcare providers are those clinicians who were directly involved in the provision of obstetric services such as delivery, antenatal care, and postnatal care. ^a^Enrolled nurses are trained for 2-3 years and are similar to licensed practical nurses in the United States ^b^Nurse assistants are trained for about 6 months and exist only in Uganda. ^c^Enrolled midwives are trained for 2-3 years. ^d^Registered nurses and ^e^registered midwives receive medical training for 3 to 4.5 years. ^f^Clinical officers receive 3 years of training. ^g^Doctors are typically trained for 5 to 7 years. Provider qualification can vary by country. ^h^Hospitals are equipped to provide comprehensive obstetric and neonatal care (CEmONC) which perform the 7 basic functions of BEmONC and two additional services: cesarean delivery and blood transfusion. ^i^Health centers are equipped to provide basic emergency obstetric and newborn care (BEmONC) which includes 7 signal functions: 1) Administration of parenteral antibiotics, 2) Administration of uterotonic drugs for active management of the third stage of labor and prevention of postpartum hemorrhage, 3) Use of parenteral anticonvulsants for the management of preeclampsia/eclampsia, 4) Manual removal of placenta, 5) Removal of retained products, 6) Assistance of vaginal delivery, and 7) Basic neonatal resuscitation. ^j^Private facility includes both for-profit and not-for profit.

## **Appendix B** – Linear regression for the association between provider and facility characteristics and confidence using knowledge and scope of practice as continuous variables among maternal healthcare providers in Uganda and Zambia, N=574.

|  | Coefficient |  | 95% CI |  | P-value |
| --- | --- | --- | --- | --- | --- |
| **Knowledge score** | 0.11 |  | [-0.03, 0.25] |  | 0.12 |
| **Scope of practice** | 0.26** |  | [0.19, 0.32] |  | 0.00 |
| **Provider characteristics** |  |  |  |  |  |
| Age | 0.00 |  | [-0.01, 0.01] |  | 0.81 |
| Age^2^ | 0.00 |  | [0.00, 0.00] |  | 0.60 |
| Female | -0.05* |  | [-0.09, -0.01] |  | 0.02 |
| Days of training in past year | 0.00 |  | [0.00, 0.00] |  | 0.25 |
| Qualification (ref. Enrolled nurse^a^) |  |  |  |  |  |
| Nurse assistant^b^ | -0.14 |  | [-0.25, -0.04] |  | 0.01 |
| Enrolled midwife^c^ | 0.08** |  | [0.04, 0.12] |  | 0.00 |
| Registered nurse^d^ | 0.03 |  | [-0.03, 0.10] |  | 0.28 |
| Registered midwife^e^ | 0.06* |  | [0.01, 0.11] |  | 0.03 |
| Clinical officer^f^ | -0.03 |  | [-0.08, 0.05] |  | 0.69 |
| Doctor^g^ | 0.14** |  | [0.06, 0.22] |  | 0.00 |
| **Facility characteristics** |  |  |  |  |  |
| Hospital^h^ (ref. Health center^i^) | -0.01 |  | [-0.04, 0.03] |  | 0.61 |
| Public facility (ref. Private facility^j^) | 0.03 |  | [0.00, 0.06] |  | 0.08 |
| Intervention district (ref. Control) | 0.03 |  | [-0.01, 0.06] |  | 0.13 |
| Zambia (ref. Uganda) | 0.01 |  | [-0.04, 0.06] |  | 0.66 |

Notes: N= 574. **p<0.01, *p<0.05. Outcome variable is a continuous variable for confidence levels. Standard errors are adjusted for clustering with facilities. Maternal healthcare providers are those clinicians who were directly involved in the provision of obstetric services such as delivery, antenatal care, and postnatal care. ^a^Enrolled nurses are trained for 2-3 years and are similar to licensed practical nurses in the United States ^b^Nurse assistants are trained for about 6 months and exist only in Uganda. ^c^Enrolled midwives are trained for 2-3 years. ^d^Registered nurses and ^e^registered midwives receive medical training for 3 to 4.5 years. ^f^Clinical officers receive 3 years of training. ^g^Doctors are typically trained for 5 to 7 years. Provider qualification can vary by country. ^h^Hospitals are equipped to provide comprehensive obstetric and neonatal care (CEmONC) which perform the 7 basic functions of BEmONC and two additional services: cesarean delivery and blood transfusion. ^i^Health centers are equipped to provide basic emergency obstetric and newborn care (BEmONC) which includes 7 signal functions: 1) Administration of parenteral antibiotics, 2) Administration of uterotonic drugs for active management of the third stage of labor and prevention of postpartum hemorrhage, 3) Use of parenteral anticonvulsants for the management of preeclampsia/eclampsia, 4) Manual removal of placenta, 5) Removal of retained products, 6) Assistance of vaginal delivery, and 7) Basic neonatal resuscitation. ^j^Private facility includes both for-profit and not-for profit.

## **Appendix C** – Linear regression for the association between provider and facility characteristics and confidence among maternal healthcare providers in the control districts in Uganda and Zambia, N=204.

|  | Coefficient |  | 95% CI |  | P-value |
| --- | --- | --- | --- | --- | --- |
| **Knowledge (ref. Knowledge score 28% - 50%)** | | | |  |  |
| 51% - 58% Knowledge score | 0.02 |  | [-0.05, 0.09] |  | 0.53 |
| 60% - 93% Knowledge score | 0.01 |  | [-0.07, 0.10] |  | 0.72 |
| **Scope of practice (ref. Scope of practice 0% - 48%)** |  |  |  |  |  |
| 50% - 65% Scope of practice | 0.06* |  | [0.01, 0.12] |  | 0.04 |
| 69% - 100% Scope of practice | 0.16** |  | [0.09, 0.22] |  | 0.00 |
| **Provider characteristics** |  |  |  |  |  |
| Age | -0.01 |  | [-0.03, 0.01] |  | 0.46 |
| Age^2^ | 0.00 |  | [0.00, 0.00] |  | 0.49 |
| Female | -0.01 |  | [-0.07, 0.05] |  | 0.81 |
| Training received in days | 0.00 |  | [0.00, 0.00] |  | 0.73 |
| Qualification (ref. Enrolled nurse^a^) |  |  |  |  |  |
| Nurse assistant^b^ | -0.22* |  | [-0.37, -0.07] |  | 0.01 |
| Enrolled midwife^c^ | 0.09* |  | [0.02, 0.16] |  | 0.01 |
| Registered nurse^d^ | 0.03 |  | [-0.07, 0.12] |  | 0.58 |
| Registered midwife^e^ | 0.06 |  | [-0.03, 0.15] |  | 0.16 |
| Clinical officer^f^ | -0.05 |  | [-0.18, 0.08] |  | 0.45 |
| Doctor^g^ | 0.21** |  | [0.10, 0.32] |  | 0.00 |
| **Facility characteristics** |  |  |  |  |  |
| Hospital^h^ (ref. Health center^i^) | 0.06 |  | [0.00, 0.12] |  | 0.07 |
| Public facility (ref. Private facility^j^) | 0.00 |  | [-0.07, 0.07] |  | 0.85 |
| **Zambia (ref. Uganda)** | 0.03 |  | [-0.02, 0.09] |  | 0.20 |

Notes: N= 204. **p<0.01, *p<0.05. Outcome variable is a continuous variable for confidence levels. Standard errors are adjusted for clustering with facilities. Maternal healthcare providers are those clinicians who were directly involved in the provision of obstetric services such as delivery, antenatal care, and postnatal care. ^a^Enrolled nurses are trained for 2-3 years and are similar to licensed practical nurses in the United States ^b^Nurse assistants are trained for about 6 months and exist only in Uganda. ^c^Enrolled midwives are trained for 2-3 years. ^d^Registered nurses and ^e^registered midwives receive medical training for 3 to 4.5 years. ^f^Clinical officers receive 3 years of training. ^g^Doctors are typically trained for 5 to 7 years. Provider qualification can vary by country. ^h^Hospitals are equipped to provide comprehensive obstetric and neonatal care (CEmONC) which perform the 7 basic functions of BEmONC and two additional services: cesarean delivery and blood transfusion. ^i^Health centers are equipped to provide basic emergency obstetric and newborn care (BEmONC) which includes 7 signal functions: 1) Administration of parenteral antibiotics, 2) Administration of uterotonic drugs for active management of the third stage of labor and prevention of postpartum hemorrhage, 3) Use of parenteral anticonvulsants for the management of preeclampsia/eclampsia, 4) Manual removal of placenta, 5) Removal of retained products, 6) Assistance of vaginal delivery, and 7) Basic neonatal resuscitation. ^j^Private facility includes both for-profit and not-for profit.

## **Appendix D** – Linear regression for the association between provider and facility characteristics and confidence among maternal healthcare providers in the intervention districts in Uganda and Zambia, N=370.

|  | Coefficient |  | 95% CI |  | P-value |
| --- | --- | --- | --- | --- | --- |
| **Knowledge (ref. Knowledge score 28% - 50%)** | | |  |  |  |
| 51% - 58% Knowledge score | -0.01 |  | [-0.06, 0.04] |  | 0.62 |
| 60% - 93% Knowledge score | 0.05* |  | [0.00, 0.10] |  | 0.03 |
| **Scope of practice (ref. Scope of practice 0% - 48%)** |  |  |  |  |  |
| 50% - 65% Scope of practice | 0.03 |  | [-0.01, 0.09] |  | 0.27 |
| 69% - 100% Scope of practice | 0.10* |  | [0.04, 0.16] |  | 0.01 |
| **Provider characteristics** |  |  |  |  |  |
| Age | 0.00 |  | [-0.01, 0.02] |  | 0.69 |
| Age^2^ | 0.00 |  | [0.00, 0.00] |  | 0.96 |
| Female | -0.07* |  | [-0.12, -0.01] |  | 0.02 |
| Training received in days | 0.00 |  | [0.00, 0.00] |  | 0.71 |
| Qualification (ref. Enrolled nurse^a^) |  |  |  |  |  |
| Nurse assistant^b^ | -0.09 |  | [-0.23, 0.05] |  | 0.20 |
| Enrolled midwife^c^ | 0.09** |  | [0.04, 0.13] |  | 0.00 |
| Registered nurse^d^ | 0.03 |  | [-0.06, 0.13] |  | 0.48 |
| Registered midwife^e^ | 0.07 |  | [-0.00, 0.14] |  | 0.05 |
| Clinical officer^f^ | 0.01 |  | [-0.07, 0.09] |  | 0.87 |
| Doctor^g^ | 0.14* |  | [0.04, 0.24] |  | 0.01 |
| **Facility characteristics** |  |  |  |  |  |
| Hospital^h^ (ref. Health center^i^) | -0.05* |  | [-0.09, -0.02] |  | 0.01 |
| Public facility (ref. Private facility^j^) | 0.03 |  | [-0.01, 0.08] |  | 0.09 |
| **Zambia (ref. Uganda)** | 0.00 |  | [-0.06, 0.06] |  | 1.00 |

Notes: N= 370. **p<0.01, *p<0.05. Outcome variable is a continuous variable for confidence levels. Standard errors are adjusted for clustering with facilities. Maternal healthcare providers are those clinicians who were directly involved in the provision of obstetric services such as delivery, antenatal care, and postnatal care. ^a^Enrolled nurses are trained for 2-3 years and are similar to licensed practical nurses in the United States ^b^Nurse assistants are trained for about 6 months and exist only in Uganda. ^c^Enrolled midwives are trained for 2-3 years. ^d^Registered nurses and ^e^registered midwives receive medical training for 3 to 4.5 years. ^f^Clinical officers receive 3 years of training. ^g^Doctors are typically trained for 5 to 7 years. Provider qualification can vary by country. ^h^Hospitals are equipped to provide comprehensive obstetric and neonatal care (CEmONC) which perform the 7 basic functions of BEmONC and two additional services: cesarean delivery and blood transfusion. ^i^Health centers are equipped to provide basic emergency obstetric and newborn care (BEmONC) which includes 7 signal functions: 1) Administration of parenteral antibiotics, 2) Administration of uterotonic drugs for active management of the third stage of labor and prevention of postpartum hemorrhage, 3) Use of parenteral anticonvulsants for the management of preeclampsia/eclampsia, 4) Manual removal of placenta, 5) Removal of retained products, 6) Assistance of vaginal delivery, and 7) Basic neonatal resuscitation. ^j^Private facility includes both for-profit and not-for profit.

## **Appendix E** - Clinical confidence questionnaire asked self-efficacy in performing twenty-seven common obstetric tasks.

| **Skill -** Prompt: For each of the following, tell me your own confidence in how good your skills are in this area—for example, are you very confident or not very confident? | Degree of Confidence  A=very confident  B=not very confident  C=I cannot perform this skill D = Does not apply |
| --- | --- |
| **A. Antenatal care** |  |
| Managing malaria in pregnancy | A B C D |
| Managing hypertension in pregnancy | A B C D |
| Managing HIV+ patients in pregnancy | A B C D |
| **B. Delivery care** |  |
| Normal childbirth | A B C D |
| Managing severe pre-eclampsia and eclampsia | A B C D |
| Monitoring labor using partograph | A B C D |
| Augmentation of labor | A B C D |
| Administered injectable antihypertensive | A B C D |
| Administered oxytocin | A B C D |
| Administered injectable antibiotics | A B C D |
| Manual removal of retained products | A B C D |
| Managing breech presentation | A B C D |
| Managing shock | A B C D |
| Active management of third stage of labor | A B C D |
| Episiotomy and repair | A B C D |
| Bimanual compression of the uterus | A B C D |
| Manual removal of placenta | A B C D |
| Repair of cervical tears | A B C D |
| Repair of perineal tears | A B C D |
| Endotracheal intubation of the mother | A B C D |
| Vacuum extraction of baby | A B C D |
| Breech delivery | A B C D |
| Manual vacuum aspiration of retained products | A B C D |
| Performing C-sections | A B C D |
| **C. Newborn care** |  |
| Newborn resuscitation (for non-breathing infant) | A B C D |
| Newborn Apgar assessment | A B C D |
| Kangaroo care | A B C D |
